# Rapid Detection and Identification of Mycotoxigenic Fungi and Mycotoxins in Stored Wheat Grain

**DOI:** 10.3390/toxins9100302

**Published:** 2017-09-25

**Authors:** Sudharsan Sadhasivam, Malka Britzi, Varda Zakin, Moshe Kostyukovsky, Anatoly Trostanetsky, Elazar Quinn, Edward Sionov

**Affiliations:** 1 Department of Food Quality and Safety, Institute for Postharvest and Food Sciences, Agricultural Research Organization, The Volcani Center, Rishon LeZion 7528809, Israel; sudharsan@volcani.agri.gov.il (S.S.); veredz@volcani.agri.gov.il (V.Z.); inspect@volcani.agri.gov.il (M.K.); anatoly@volcani.agri.gov.il (A.T.); elazar@volcani.agri.gov.il (E.Q.); 2 National Residue Control Laboratory, Kimron Veterinary Institute, Bet Dagan 50250, Israel; malkab@moag.gov.il

**Keywords:** toxigenic fungi, aflatoxins, fumonisins, deoxynivalenol, wheat grains, multiplex PCR, LC/MS/MS

## Abstract

This study aimed to assess the occurrence of toxigenic fungi and mycotoxin contamination in stored wheat grains by using advanced molecular and analytical techniques. A multiplex polymerase chain reaction (PCR) strategy was established for rapid identification of mycotoxigenic fungi, and an improved analytical method was developed for simultaneous multi-mycotoxin determination in wheat grains by liquid chromatography-tandem mass spectrometry (LC/MS/MS) without the need for any clean-up. The optimized multiplex PCR method was highly specific in detecting fungal species containing species-specific and mycotoxin metabolic pathway genes. The method was applied for evaluation of 34 wheat grain samples collected from storage warehouses for the presence of mycotoxin-producing fungi, and a few samples were found positive for *Fusarium* and *Aspergillus* species. Further chemical analysis revealed that 17 samples contained mycotoxins above the level of detection, but only six samples were found to be contaminated over the EU regulatory limits with at least one mycotoxin. Aflatoxin B_1_, fumonisins, and deoxynivalenol were the most common toxins found in these samples. The results showed a strong correlation between the presence of mycotoxin biosynthesis genes as analyzed by multiplex PCR and mycotoxin detection by LC/MS/MS. The present findings indicate that a combined approach might provide rapid, accurate, and sensitive detection of mycotoxigenic species and mycotoxins in wheat grains.

## 1. Introduction

Wheat grain and associated by-products are important sources of energy and protein for humans and all classes of farm animals. When grains are colonized by moulds there is a significant risk of contamination with mycotoxins, which are toxic chemical products, formed as secondary metabolites by these fungi. Many species of *Fusarium*, *Aspergillus*, *Penicillium*, and *Alternaria* are not only recognised as plant pathogens but are also sources of important mycotoxins of concern in relation to animal and human health [1]. Traditionally, toxigenic fungi in crops have been divided into two distinct classes: ‘field fungi’ (or plant pathogens), which invade and produce their toxins before harvest; and ‘storage’ (or saprophytic) fungi, which become a problem after harvest. However, the original source of both of these classes of fungi is in the field [2]. Moreover, some fungi might belong to both classes and colonize grains before and after harvest. *Aspergillus flavus*, for example, is associated with *Aspergillus* infections of grain crops in the field; it also contaminates stored grains when prevailing abiotic factors (such as temperature and water activity) are favourable [3,4]. Interactions between environmental stress factors, such as water activity and temperature, may have an influence on growth, expression of biosynthetic regulatory genes and mycotoxin production by mycotoxigenic fungal species [5,6]. Good postharvest practices avoiding high temperatures and rapid drying of grain can avoid a decrease in the grain quality and reduce the health risk due to mould growth and potential toxin contamination [6]. Poor postharvest management can lead to rapid deterioration in quality, with severe decreases in germinability and nutritional value of stored grain, possibly accompanied by undesirable fungal contamination and, consequently, toxin production [7]. The most frequently detected mycotoxins in wheat grain are deoxynivalenol (DON), fumonisins (Fs), and zearalenone (ZEN), produced by *Fusarium* species, and aflatoxins (AFs) and ochratoxin A (OTA) produced by *Aspergillus* and *Penicillium* species, respectively [8]. Due to their wide range of physical and chemical properties, mycotoxins are stable chemical compounds which cannot be destroyed during most food processing operations. The Food and Agricultural Organization (FAO) of the United Nations estimated that each year approximately 25% of the world’s crops are contaminated by mycotoxins, which cause annual losses of around one billion metric tons of food products. Timely assessment of these contaminants and identification of the main toxicogenic fungal species are important, not only for assessing food quality, but also for the development of control strategies for ensuring food safety [9]. Surveillance and monitoring for mycotoxigenic fungi and mycotoxins becomes critical for maintaining a high quality of grains and grain products in indoor storage facilities.

Molecular approaches, such as PCR, can serve as good alternatives to conventional methods for detection of mycotoxigenic fungi. Multiplex PCR assays that simultaneously amplify numbers of species-specific genes, and/or structural or regulatory genes involved in mycotoxin biosynthesis pathways have been successfully applied to the detection of mycotoxigenic fungi in a variety of foods and feeds [10,11,12,13]. However, there have been only a few examples of differentiation of mycotoxin-producing fungi in wheat grains by means of multiplex PCR. Wang and co-workers developed a multiplex PCR assay for simultaneous detection of genes involved in biosynthesis of type B trichothecene mycotoxins that are found in wheat grain [14], with emphasis on the genetic identification of certain mycotoxin chemotypes of *Fusarium* species. Further work is now needed in order to develop a sensitive PCR assays for detection of a wide range of mycotoxigenic fungi in cereal grains.

Traditionally, mycotoxin analyses are mainly performed by means of high-performance liquid chromatography after clean-up in a solid phase extraction/immunoaffinity column [15,16]. These methods normally enable the determination of only single classes of mycotoxins, including a limited number of target analytes, increasing the cost and time of the analysis due to labour-intensive sample preparation. In order to improve the sensitivity of mycotoxin detection and to reduce the cost of the analyses it would be preferable to determine as many mycotoxins as possible by routine analysis in different types of matrices in one single extraction. Furthermore, multi-mycotoxin determination methods are required because of the co-occurrence of several mycotoxins in various commodities [17]. The complexity of the wheat grain matrix, as well as the wide range of physical and chemical properties of mycotoxins, necessitate selective and sensitive detection techniques for co-occurring toxins. Liquid chromatography/mass spectrometry (LC/MS), and particularly LC coupled to tandem mass spectrometry (LC/MS/MS) have become very popular in recent years for mycotoxin analysis. However, a multi-toxin sample extraction and preparation can be challenging due to matrix effects and chemical diversity of the analytes. Solid phase extraction methodology has been developed for analysis of a number of mycotoxins in foodstuffs [18,19,20]. The major drawback of this procedure is that it is time consuming, especially when large numbers of samples need to be analysed [21]. Immunoaffinity columns, for clean-up purposes, have become increasingly popular in recent years due to the high degree of selectivity and specificity [22,23,24]. The fact that these columns are only used once and their relative high costs are major disadvantages. The use of various modifications of the QuEChERS methodology (quick, easy, cheap, effective, rugged, and safe approach) has also been recently introduced in multi-mycotoxin analysis [25]; however, the efficiency of the method for eliminating matrix effects has yet to be determined. In the current study we developed a new low-cost, rapid, and simple extraction and analysis method for multi-toxin detection in stored wheat grains. Considering the chemical diversity of the analytes, crude sample extracts were injected into the LC/MS/MS system without further purification. The method was successfully applied for rapid and simultaneous determination of 10 mycotoxins in stored wheat grains. To the best of our knowledge, this is the first report on evaluation and optimization of a reliable multiplex PCR assay and LC/MS/MS multi-method for simultaneous detection and accurate determination of the critically important mycotoxigenic fungi and mycotoxins, respectively, in wheat grains collected from storage warehouses in Israel. This combined approach will enable to evaluate the mycotoxicological risk of stored wheat grains, to minimize economic losses and reduce the hazard to animal and human health.

## 2. Results and Discussion

### 2.1. Specificity and Sensitivity of Multiplex PCR

In this study, a multiplex PCR assay was used to facilitate simultaneous detection of a wide range of potentially mycotoxin-producing fungi in wheat grain storage warehouses in Israel. Five sets of four or five species-specific primer pairs were assembled for the molecular identification of the fungal species most frequently found in stored wheat grain [26]. They comprised seven *Aspergillus* spp., nine *Fusarium* spp., and five *Penicillium* spp. (Table 1).

**Figure 1:**
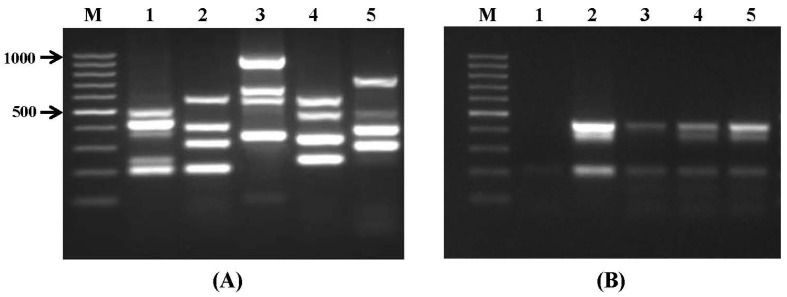
Specificity and sensitivity of multiplex PCR assays using species specific primers. (**A**) Specificity test. Lanes: M-100 bp DNA ladder; 1: primer set I; 2: primer set II; 3: primer set III; 4: primer set IV; 5: primer set V; and (**B**) sensitivity test. Lanes: 1: negative control; 2: positive control (wheat sample inoculated by *A. parasiticus* NRRL 6111, *A. fumigatus* NRRL 62427, *A. carbonarius* NRRL 368 and incubated for 48 hrs); 3–5 (represent samples inoculated by *A. parasiticus* NRRL 6111, *A.fumigatus* NRRL 62427, *A. carbonarius* NRRL 368 without incubation): 3: 10^4^ spores/g; 4: 10^5^ spores/g; and 5: 10^6^ spores/g.

The specificity of all the species-specific primers was assessed by performing multiplex PCR on the genomic DNA of standard fungal isolates, which resulted in complete amplification of all target genes (Figure 1A). In addition, three sets of four to six primers were assembled, to amplify the genes associated with mycotoxin biosynthesis, in order to detect mycotoxigenic fungi (Table 2). The annealing temperature of each primer group for amplification of the target genes was set by using temperature gradient conditions. The specificity of the developed multiplex PCR assay was confirmed; it provided good discrimination among the tested species, as well as between mycotoxin-producing and non-producing strains. In addition, none of the primers showed cross-reactions with the species amplified by the respective other primers.

The PCR-based detection of microorganisms in food and feed, as well as in biological samples, is always challenging because of the variety of species that need to be determined, or the presence of substances that inhibit the PCR or reduce its amplification efficiency, either of which can cause complete reaction failure, leading to false negative results or reduced sensitivity of specific detection of the mycotoxin-producing moulds [27]. However, in the present study, the newly-developed assays have advantages over previously-reported multiplex PCRs in terms of the broad range of mycotoxigenic fungi detected in a few single assays, which could detect all target fungal species with higher specificity (Figure 1A). To determine the minimum amount of fungal template necessary to obtain visible amplification products, the multiplex PCR assays were carried out with serial dilutions of fungal genomic DNA. The detection limit for purified genomic DNA to produce a visible band on an ethidium bromide-stained agarose gel was determined to be 100 pg for all strains tested. When the sensitivity of the multiplex PCR was evaluated by artificial inoculation of wheat grain samples with known amounts of *Aspergillus* spp. conidia, the detection limit for DNA of each species, extracted directly from grains, was 1 × 10^4^ spores/g in samples with no incubation (Figure 1B). The sensitivity of the PCR reaction might be influenced by various components of biological matrices, such as fats, or phenolic and polysaccharide compounds, which can reduce the purity of the extracted DNA [10,28,29]. In some previous studies, an enrichment step was performed that involved incubation of contaminated food samples for a certain period of time, to improve the sensitivity of fungal-specific PCR assays [8,12,30]. Nevertheless, in the present study, the higher efficacy of the assay was achieved by use of an efficient DNA extraction method and optimized PCR conditions, and it resulted in an improvement of at least one log of sensitivity, compared with that reported in previous studies [10,31].

### 2.2. Application to Stored Wheat Grain Samples

**Figure 2:**
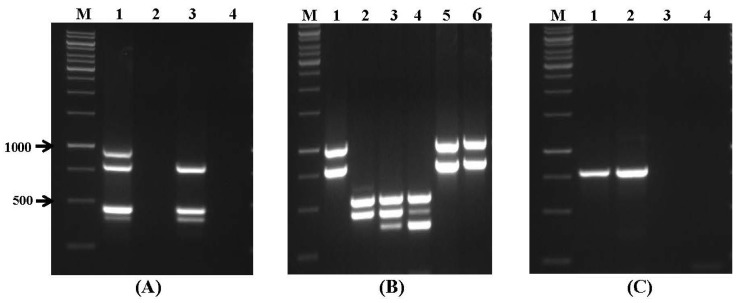
Specificity of multiplex PCR assays using sets of primers for mycotoxin biosynthetic genes. (**A**) Primer set VI. Lanes: M: 1 kb DNA ladder; 1: *A. parasiticus* NRRL 6111; 2: *A. flavus* NRRL 3518; 3: *A. flavus* SS1; 4: *A. flavus* SS2; (**B**) primer set VII. Lanes: 1: *F. verticillioides* NRRL 25457; 2: *F. sporotrichioides* NRRL 3299; 3: *F. culmorum* NRRL 13320; 4: *F. graminearum* NRRL 3376; 5: *F. verticillioides* SS4; 6: *F. verticillioides* SS5; and (**C**) primer set VIII. Lanes: 1: *P. verrucosum* NRRL 965; 2: *P. viridicatum* NRRL 5571; 3: *A. carbonarius* NRRL 368; and 4: *A. ochraceus* NRRL 35018.

In light of the high importance of wheat grain storage within the marketing, distribution, and food security system, the high quality and safety of this product need to be ensured. In order to characterize mycotoxin-producing fungi by multiplex PCR, sets of the primers directed to the structural and regulatory genes involved in biosynthesis of aflatoxins (*aflR1*, *nor1*, *avfA*, *ver1*), *Fusarium* toxins (*fum1*, *fum13*, *tri5*, *tri6*, *zea*), and OTA (*pks*, *otanps*) were tested by using DNA extracted from various species of *Aspergillus*, *Fusarium*, and *Penicillium*, respectively (Figure 2A–C). The obtained results clearly show the presence of specific fragments amplified from DNA of isolates with potential mycotoxin-producing abilities. Some *Aspergillus* strains (*A. flavus* NRRL 3518, *A. flavus* SS2, *A. carbonarius* NRRL 368, and *A. ochraceus* NRRL 35018) did not show amplification in any target genes (Figure 2A,C). The probability of a particular toxin being produced can be predicted according to the presence or absence of an amplification product, but the effective biosynthesis of the toxin remains to be confirmed by analytical chemistry analysis [32,33].

**Figure 3:**
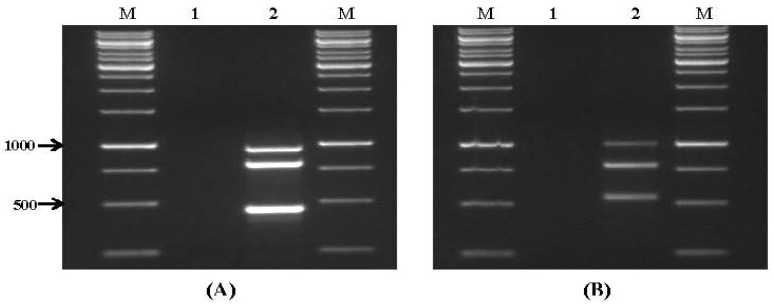
Specificity of multiplex PCR assays in artificially contaminated wheat grain samples. (**A**) Primer set VI. Lanes: M: 1 kb DNA ladder; 1: negative control (no inoculation); 2: wheat sample artificially inoculated with *A. parasiticus* NRRL 6111; and (**B**) primer set VII. Lanes: 1: negative control (no inoculation); 2: wheat sample artificially inoculated with *Fusarium* spp.

Primer sets for genes related to aflatoxin and *Fusarium* toxin biosynthesis were also tested on DNA samples obtained from wheat grain artificially infected with *A. parasiticus* NRRL 6111 (Figure 3A) and *Fusarium* spp. (Figure 3B). These primers enabled us to identify potential aflatoxigenic, fumonisin- and trichothecene-producing isolates in wheat grains. Primer sets for genes related to aflatoxin and *Fusarium* toxin biosynthesis were also tested on DNA samples obtained from wheat grain artificially infected with *A. parasiticus* NRRL 6111 (Figure 3A) and *Fusarium* spp. (Figure 3B). These primers enabled us to identify potential aflatoxigenic, fumonisin-, and trichothecene-producing isolates in wheat grains.

Since wheat grains could easily be contaminated with a variety of mycobiota before harvest, and during postharvest handling and storage, multiplex PCR assays were applied; a total of 34 wheat grain samples collected from eight storage warehouses were tested for the presence of genes involved in the biosynthesis of mycotoxins. The samples were examined directly by using this method, without a fungus isolation and incubation step. Among the 34 grain samples 22 showed positive signals when mycotoxin gene-based primer sets were used (Table 3).

**Figure 4:**
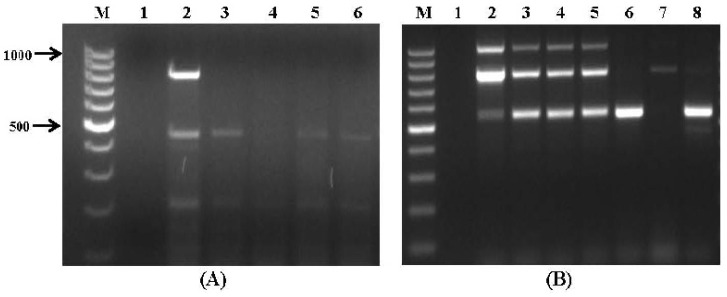
Multiplex PCR using DNA isolated from stored wheat grain. (**A**) Primer set VI. Lanes: 1: negative control; 2–6: wheat grain naturally contaminated by aflatoxicgenic *Aspergillus* spp.; and (**B**) primer set VII. Lanes: M: 100 bp DNA ladder; 1: negative control; 2–8: wheat grain naturally contaminated by toxicgenic *Fusarium* spp.

Figure 4 shows some of the results obtained when a multiplex PCR was applied to DNA extracted from wheat samples collected from two storage warehouses in northern Israel; in this case amplification products were obtained, indicating the presence of potentially aflatoxigenic *Aspergillus* isolates (Figure 4A) and toxigenic *Fusarium* spp. (Figure 4B). There was no amplification in the clean, uncontaminated sample that was used as a negative control. As noted above, relatively moderate amounts of fungal contamination (at least 10^4^ spores/g) could be detected by multiplex PCR. Yet, these results suggest that this method can be used as a diagnostic tool, which may enable the rapid and cost-effective detection of potential mycotoxigenic fungi in stored wheat grain.

### 2.3. Development of the Analytical Method

The LC/MS/MS multi-toxin method was optimized for the detection and quantification of AFB_1_, AFB_2_, AFG_1_, AFG_2_, OTA, ZEN, DON, FB_1_, FB_2_, and T-2 toxins. Due to the large number and the chemical diversity of the analytes, the application of an appropriate combination of solvents is required for extraction procedure during the development of a multi-toxin method. Different mixtures of water and organic solvents (such as methanol and acetonitrile) with or without the addition of acetic acid were tested. The best recovery and specificity values were obtained using a water/methanol (25:75 *v*/*v*) mixture for the extraction of the 10 mycotoxins from wheat grain samples. In contrast to the majority of the studies that have described an additional clean-up procedure [34,35,36,37], in the current study the developed multi-toxin method allows direct injection of the crude extracts to LC/MS/MS system with no further clean-up and with sufficient sensitivity and selectivity to detect and accurately quantify mycotoxin contents in wheat grains at levels lower than the maximum residue limit (MRL, value fixed by the legislation). The mycotoxins of interest in this study belong to different groups in terms of chemical properties. Therefore, the mobile phase composition was also an important factor which influenced the signal intensity and, thus, the sensitivity of each compound. Several combinations of mobile phase were tested including formic acid, acetic acid, ammonium formate, ammonium acetate, methanol, and acetonitrile. Finally, ammonium acetate acidified with acetic acid in combination with methanol was chosen as the best compromise for multi-mycotoxin analyses. The gradient rate was optimized to avoid co-eluting compounds from the matrix (especially for AFG_2_ qualifier ion). Mycotoxin validation results fulfilled the acceptance criteria in terms of accuracy, linearity, selectivity, and precision. The calibration levels, calculated concentration (for accuracy), precision (RSD%), limit of quantification (LOQ) and limit of detection (LOD) are summarized in Table 4. Calibration curves were linear (R2 > 0.995) for all compounds and the selectivity was acceptable for all compounds (interfering peaks were <30% of LOQ).

### 2.4. Mycotoxin Detection in Stored Wheat Grain

The results of the multiplex PCR assays were found to be in good agreement with those obtained by chemical analysis of the samples (Table 5), which were subjected to mycotoxin screening by LC/MS/MS. The LODs values obtained in the present study ranged from 0.1 ppb for aflatoxins to 3.2 ppb for DON. Overall, 17 wheat samples were positive for mycotoxins above their LODs; however, only six analysed samples were contaminated over the EU regulatory limits with at least one mycotoxin [38]. AFB_1_ was detected in four postharvest wheat samples at levels above the EU regulatory limit of 2 ppb in grains for human consumption (Table 5); these samples were found to be contaminated with *A. flavus* possessing aflatoxin biosynthesis genes (Figure 2A) and *A. flavus*-specific aspergillopepsin *pepO* gene (Figure S1A).

Molecular analysis of the grain samples revealed amplification of the *ver1* and/or *aflR1* genes (Figure 4A), which are associated with aflatoxin biosynthesis. A PCR-based assay of *Fusarium* mycotoxin biosynthesis genes showed the amplification of *fum1* and *fum13* genes associated with fumonisin biosynthesis in four wheat samples (Figure 4B, lanes 2–5; samples #18–21). Among the strains isolated from the samples *F. verticillioides* was identified by morphological and molecular characterization as the predominant species, harbouring the *fum1* and *fum13* genes (Figure 2B). The LC/MS/MS analysis clearly indicated the presence of FB_1_ and FB_2_ toxins in the samples with high FB_1_ contamination (2.34 ppm) in stored grain sample #18 (Table 5). Another two wheat DNA samples (#22 and #24) showed amplification of *tri6* gene involved in trichothecene biosynthesis, according to the results of the multiplex PCR assay (Figure 4B; lanes 6 and 8). When these DNA samples were tested against *Fusarium* species-specific sets of primers the 570-bp amplification fragment was obtained, which indicated the presence of *F. culmorum* (Figure S1B). The grain samples were cultured to confirm this identification and were found to be contaminated predominantly with *F. culmorum* that exhibited DON-producing abilities. Type B trichothecene DON, also known as vomitoxin, was detected and quantified by LC/MS/MS in both samples; its concentration exceeded the regulatory limit in sample #24 (1.74 ppm; Table 5). It has been reported previously that *F. verticillioides* and *F. culmorum* were isolated from various corn growing areas in Israel [39,40]; because of the rise in average temperatures caused by climate change, these species are now frequently reported as the main agents of *Fusarium* diseases of cereals in the Mediterranean region, especially in wet years [41,42,43,44]. Moreover, *F. verticillioides* and *F. culmorum* are also known as postharvest pathogens, especially on freshly-harvested grain that has not been dried or stored properly [7,45]. Further research is needed to study the relationship between interacting environmental stress factors (such as temperature and water availability) and the activity of key mycotoxigenic fungi, occurring at postharvest stage of wheat grains, for better understanding the conditions which represent higher risks from mycotoxin production.

A strong correlation was found between LC/MS/MS analysis of three stored grain samples (#4–#6), in which FB_1_ and FB_2_ were present at values below the regulatory guidelines (Table 5), and an established multiplex PCR assay of DNAs extracted from the same wheat samples, resulted in specific amplification of target genes *fum1* and *fum13*. However, some differences were observed between molecular and analytical chemistry results: in particular, samples #18–#21, in addition to *fum1* and *fum13* fragments, showed amplification of the *tri6* gene by multiplex PCR (Figure 4B), whereas the production of only fumonisins was observed by multi-mycotoxin LC/MS analysis. This could be because a lack of proper environmental conditions might have inhibited the expression of specific toxin metabolic pathway genes [11]. Furthermore, the PCR assay might yield false positive results because of difficulties in detecting mutations outside the primers’ targeted gene sequence region [46]. Nevertheless, some researchers have pointed out that, for diagnostic purposes of screening food and feed samples, false positive results in the detection of mycotoxigenic fungi are more acceptable than false negatives [10,47]. The use of internal amplification controls can prevent false negatives that might be caused by PCR inhibitors; these controls are often used to rule out failure of amplification in cases where the target sequence is not detected [48]. In the present study the nucleic acid amplification tests did not include internal controls because of the high efficacy of the assays and the positive correlation between the multiplex PCR and LC/MS/MS results (correlation coefficient of 0.99). The need to use internal controls should be determined on a case-by-case basis, because development and implementation of such controls can be difficult, and they can impair assay sensitivity [49]. Moreover, if the reaction failure rate is found to be ≤2% during test verification, routine use of an internal control is not necessary [50].

In conclusion, a relatively small number of stored wheat grain samples were found to be contaminated, mainly by *Fusarium* and *Aspergillus* spp. Co-occurrence of different mycotoxins produced by these fungi was observed, although most of the toxins were detected at very low levels (Table 5), as mandated by international regulatory standards. Nevertheless, the potential presence of opportunistic human pathogens, such as *A. fumigatus*, in stored wheat grain may represent a high risk of lung infection (pulmonary aspergillosis) to farm workers who have a compromised immune system. The current study might have important implications for keeping mycotoxin-producing fungi and mycotoxins out of the food-supply chain and reducing the hazard to human health. The strong correlation between multiplex PCR and LC/MS/MS results obtained in the present study demonstrated a rapid, accurate, and sensitive means for detection of mycotoxigenic species and mycotoxins in wheat grains. Combinations of molecular and analytical procedures in food safety laboratories might serve as a reliable diagnostic means for the rapid screening of large numbers of samples. In addition, such an approach could be very useful for obtaining information about the potential toxigenicity of fungal species and their concomitant mycotoxins that might contaminate wide ranges of agricultural and food products.

## 3. Materials and Methods

### 3.1. Strains and Media

Standard fungal strains (Table S2) were obtained from the USDA Agricultural Research Service Culture Collection (Northern Regional Research Laboratory, Peoria, IL, USA); they were stored in 25% glycerol at −80 °C until use and then were maintained on PD (0.4% potato starch, 2% dextrose) agar plates at 30 °C. The strains were grown in YPD liquid medium (1% yeast extract, 2% peptone, 2% dextrose) for genomic DNA extraction.

### 3.2. Wheat Grain Samples

Samples were taken from eight wheat grain storage warehouses in various parts of Israel, 3–6 months after harvest. In each warehouse 1-kg aliquots of grain, destined for human consumption, were collected from the front face and the centre, at points located 1 m in horizontal depth within the grain mass, and from areas close to the walls. Grain temperature and moisture content were in the ranges of 27–33 °C and 10.5–12.9%, respectively. The collected samples were kept in sterile plastic bags during transport to the laboratory and on the same day a 100-g aliquot from each thoroughly-mixed sample was frozen in liquid nitrogen, lyophilized, and milled into a fine powder with a grain grinder. The samples were stored at 4 °C pending analysis.

### 3.3. Reagents and Solvents

All common chemicals and solvents used for DNA extraction were purchased from Sigma-Aldrich and Bio-Lab (Jerusalem, Israel), respectively, unless otherwise specified.

Mycotoxin standards were purchased from Fermentek (Jerusalem, Israel). Methanol and acetonitrile (both LC gradient grade) were obtained from J.T. Baker (Deventer, The Netherlands); ammonium acetate and acetic acid (MS grade) were obtained from Fisher Scientific (Fair Lawn, NJ, USA). Ultrapure water was obtained using a Micropure purification system (Thermo Scientific, Langenselbold, Germany).

### 3.4. DNA Extraction

Fungal genomic DNA was isolated from lyophilized mycelial mats grown overnight in YPD medium. The DNA was extracted using a lysis buffer containing hexadecyltrimethylammonium bromide (CTAB). Lyophilized mycelial mats were pulverized with 5 mL of 3-mm-diameter glass beads in a disposable 50-mL conical centrifuge tube. Ten millilitres of DNA extraction buffer (l.0 M Tris/HCl, pH 7.5; 1% (*w*/*v*) CTAB; 5 M NaCl; 0.5 M EDTA; 1% (*v*/*v*) 2-mercaptoethanol; and proteinase K at 0.3 mg/mL) were added to the powdered mycelia and mixed gently, and the mixture was incubated at 65 °C for 30 min. The extracts were cooled prior to the addition of an equal volume of chloroform, gently mixed and centrifuged at 6000 rpm for 10 min. The aqueous supernatant was recovered and the nucleic acids were precipitated with an equal volume of 2-propanol. Gentle mixing resulted in the formation of high-molecular-mass DNA, which was precipitated by centrifugation at 4800× *g* for 5 min. The DNA was resuspended in TE buffer solution (Tris-EDTA, pH 8.0) containing RNase A at 10 µg/mL, and further purified by phenol-chloroform extraction (A260/A280 ratio of 1.8–2.0). Finally, the DNA was precipitated with 100% ethanol containing 3 M sodium acetate, rinsed in 70% (*v*/*v*) ethanol and resuspended in TE buffer. The DNA extraction from wheat grain samples was performed as described above. The purity of the extracted DNA was assayed with an ND-2000 spectrophotometer (Thermo Scientific, Wilmington, DE, USA).

### 3.5. Multiplex PCR

In order to optimize the multiplex PCR assay for direct detection of mycotoxigenic fungal species in naturally infected wheat grain samples, primers used previously for species-specific detection and for amplification of genes involved in mycotoxin biosynthesis (Table 1, Table 2 and Table S2) were tested for referenced fungi and confirmed by performing monoplex PCR. The multiplex PCR was standardized by empirically varying critical factors that affect multiplexing, such as primer concentrations, template amount, and annealing temperature. Eight different sets of 4–6 pairs of primers were combined for multiplex PCR, with annealing temperature and amplicon size taken into account. Multiplex PCR was performed in a 25-µL reaction mix containing 20–50 ng of each genomic DNA as a template, 2 × multiplex PCR master-mix with 3 mM MgCl_2_ (QIAGEN, Hilden, Germany) and each primer at 0.2 µM. The PCR sequence comprised: initial heat activation of DNA polymerase at 95 °C for 15 min, followed by 35 cycles of denaturation at 94 °C for 30 s, annealing at 55–60 °C (Table 1 and Table 2) for 90 s, extension at 72 °C for 90 s, and a final extension at 72 °C for 10 min. PCR products were electrophoresed on a 1% agarose gel with a 100-bp DNA size marker at 96 V for 1 h.

### 3.6. Multiplex PCR with Artificially Contaminated Wheat Grain Samples

Validation and sensitivity assessment of the method were carried out by inoculation of 2 g of sterile wheat grain samples with the individual spore suspensions (10^4^–10^6^ spores/g) of *A. carbonarius*, *A. fumigatus*, and *A. parasiticus*; an uninoculated sample was used as a negative control. DNA was isolated from the samples as described above, and subjected to multiplex PCR assay.

### 3.7. Isolation of Fungal Species from Wheat Grain

The wheat samples were analysed for presence of potentially mycotoxigenic fungi. The fungi were obtained by direct plating of wheat seeds from each sample onto PDA media supplemented with chloramphenicol at 10 µg/mL, incubated at 28 °C for three days, and identified by morphological analysis and sequencing of ribosomal DNA internal transcribed spacers (ITS). The ITS regions 1 and 2 of ribosomal DNA were used to compare the ITS1-ITS2 nucleotide sequences. The universal ITS primers, ITS1 (5′-TCCGTAGGTGAACCTGCGG-3′) and ITS4 (5′-TCCTCCGCTTATTGATATGC-3′) were used and the ITS regions were amplified selectively by PCR. The sequence of nucleotide alignments obtained was referenced against the GeneBank database [51] with the nucleotide BLAST program.

### 3.8. Preparation of Mycotoxin Standard Solutions

Individual stock standard solutions (1 mg/mL) of aflatoxins B_1_, B_2_, G_1_, and G_2_ (AFB_1_, AFB_2_, AFG_1_, AFG_2_), OTA, ZEN, DON, fumonisins B_1_ and B_2_ (FB_1_, FB_2_), and T-2 toxin (T-2) were prepared in methanol. Multi-toxin working standard solutions with a series of toxin concentrations were prepared by dilution of the stock solutions of the analytes in methanol. All solutions were stored at –20 °C and were brought to room temperature before use.

### 3.9. Sample Preparation for Mycotoxin Analysis

Five grams of the ground sample material were mixed with 20 mL of 25:75 (*v*/*v*) water/methanol and placed in an orbital shaker for 30 min. After centrifugation at 8500× *g* for 15 min, 5 mL of the supernatant were transferred to a 15-mL glass tube and evaporated under a stream of nitrogen gas at 50 °C. The dry residue was reconstituted with 0.25 mL of a 95:5 (*v*/*v*) water/methanol mixture and centrifuged for 10 min at 17,000× *g*, at 4 °C; the supernatant was used directly for the analysis. Samples, in which the concentration exceeded the highest level of calibration, were diluted and re-injected.

### 3.10. Instrumentation for Mycotoxin Analysis

Detection and quantification of mycotoxins were performed with high-performance liquid chromatography coupled with tandem mass-spectrometry (LC/MS/MS). Chromatographic separation was carried out using Nexera X2 UHPLC (Shimadzu, Tokyo, Japan) equipped with 100 × 2.1 mm, 2.6 µm Kinetex C_18_ column, (Phenomenex, Torrance, CA, USA). The column was maintained at 40 °C and the injection volume was 2 µL. The mobile phase consisted of 2.5 mM ammonium acetate acidified with 0.1% acetic acid (A), and methanol (B). The methanol (B) concentration was raised gradually from 5% to 95% within 8 min, brought back to the initial conditions at 9 min, and allowed to stabilize for 3 min. The mobile phase was delivered at a flow rate of 0.4 mL/min. The LC system was coupled with API 6500 hybrid triple quadrupole/linear ion trap mass spectrometer (Sciex, Concord, ON, Canada), equipped with a turbo-ion electrospray (ESI) ion source. The mass-spectrometer was operated in scheduled multiple reaction monitoring (sMRM) in both positive and negative mode within a single run. Positive polarity was applied for all analytes except for DON and ZEA. Precursor/quantifier/qualifier ions are specified in Table 4. Source temperature was set at 350 °C, ion-spray voltages at −4500 V (negative mode) and 5000 V (positive mode), curtain gas at 35 arbitrary units (au), nebulizer gas at 60 au, and turbo gas at 40 au.

### 3.11. Validation of Analytical Parameters

Validation of the method was carried out in wheat matrix, according to Commission Regulation (EC) No 401/2006 [52]. Three non-contaminated wheat grain samples were spiked with multi-mycotoxin standard solutions at three concentration levels. The calibration levels are specified in Table 4. Extraction and analysis were performed as described above. The spiking experiments were performed in triplicate at three different time points. Validation parameters, such as precision, accuracy, LOD, LOQ, and specificity, were determined.

## Figures and Tables

**Table 1:** Sets of species-specific primers used in this study ^1^.

Set No.	Species	DNA/Gene Target	Amplicon Size (bp)	Annealing Temperature
I	*A. tubingensis*	*caM*	505	
	*A. parasiticus*	*caM*	430	
	*A. carbonarius*	*caM*	371	60 °C
	*A. fumigatus*	*pep*	250	
	*A. flavus*	*pepO*	200	
II	*F. culmorum*	RAPD ^2^ marker	570	
	*F. graminearum*	RAPD marker	400–500	
	*F. sporotrichioides*	*tri13*	332	55 °C
	*F. poae*	RAPD marker	220	
III	*F. avenaceum*	RAPD marker	920	
	*F. verticillioides*	*caM*	578	
	*A. niger*	*caM*	357	55 °C
	*F. solani*	AFLP	175	
IV	*F. proliferatum*	*caM*	585	
	*P. expansum*	*IDH*	480	
	*F. oxysporum*	*ITS*	340	55 °C
	*P. digitatum*	*Cyp*51	250	
V	*P. verrucosum*	*Otanps*	750	
	*P. paneum*	*IDH*	482	
	*A. terreus*	Topoisomerase II	386	60 °C
	*P. roqueforti*	ITS1-5.8S-ITS2	300	

^1^ See Table S1 for detailed description of the primers used in the study; ^2^ RAPD: random amplified polymorphic DNA.

**Table 2:** Primer sets for genes involved in mycotoxin biosynthesis ^1^.

Set No.	Mycotoxin	Gene Target	Amplicon Size (bp)	Annealing Temperature
VI	Aflatoxins	*avfA*	950	
		*aflR1*	798	
		*ver1*	452	58 °C
		*nor1*	397	
VII	Fumonisin/Trichothecene/Zearalenone	*fum13*	988	
		*fum1*	798	
		*tri6*	546	55 °C
		*tri5*	450	
		*pks13*	351	
VIII	Ochratoxin A	*otanps*	788	
				58 °C


^1^ See Table S1 for detailed description of the primers used in the study.

**Table 3:** Detection of mycotoxin biosynthetic pathway genes by multiplex PCR in stored wheat grain samples.

Wheat Sample #	Mycotoxin Biosynthetic Gene Specific Primer Sets										
	Aflatoxins (Set No. VI)				*Fusarium* Toxins (Set No. VII)					OTA (Set No. VIII)	
	*aflR1*	*nor1*	*avfA*	*ver1*	*fum1*	*fum13*	*tri5*	*tri6*	*pks13*	*pks*	*otanps*
1–3	-	-	-	-	-	-	-	-	-	-	-
4	+	-	-	+	+	+	-	-	-	-	-
5	+	-	-	+	+	+	-	-	-	-	-
6	+	-	-	+	+	+	-	+	-	-	-
7	-	-	-	+	-	-	-	-	-	-	-
8	-	-	-	+	-	-	-	-	-	-	-
9	-	-	-	+	-	-	-	-	-	-	-
10	-	-	-	-	-	-	-	-	-	-	-
11	-	-	-	-	+	+	-	-	-	-	-
12–15	-	-	-	-	-	-	-	-	-	-	-
16	-	-	-	+	+	+	-	-	-	-	-
17	-	-	-	-	-	-	-	-	-	-	-
18	+	-	-	+	+	+	-	-	-	-	-
19	+	-	-	+	+	+	-	-	-	+	-
20	+	-	-	+	+	+	-	-	-	-	-
21	+	-	-	+	+	+	-	-	-	-	-
22	-	-	-	-	-	-	-	+	-	+	-
23	-	-	-	-	-	-	-		-	+	-
24	-	-	-	-	-	-	-	+	-	+	-
25	-	-	-	+	-	-	-	-	-	-	-
26	+	-	-	+	+	+	-	-	-	-	-
27–34	-	-	-	-	-	-	-	-	-	-	-

“+” detected; ”-“ not detected

**Table 4:** Summary of LC/MS/MS validation results.

Compound	Precursor Ion m/z	Quantifier (Qualifier) Ions m/z	Calibration Level (ppb)	Calculated Concentration ppb (±SD) ^1^	RSD (%) ^2^	LOQ (LOD) ppb
DON	355 [M + CH_3_COO]^−^	295 (265)	40	41 (3.6)	8.9	11.4 (3.2)
			80	82.5 (4.8)	5.9	
			640	634.6 (27.4)	4.4	
ZEN	317 [M − H]^−^	131 (175)	20	21.8 (4.9)	22.5	6.3 (1.6)
			40	36.7 (2.4)	6.4	
			320	300 (22.5)	7.5	
T-2	484 [M + NH_4_]^+^	215 (185)	40	37.6 (5.9)	15.7	8.1 (2.4)
			80	77.5 (8.8)	11.4	
			640	650.2 (18.9)	2.9	
OTA	404 [M + H]^+^	239 (102)	2	2.1 (0.2)	10.2	0.8 (0.2)
			4	4.3 (0.25)	5.7	
			32	32.8 (2.95)	9.2	
FB_1_	722 [M + H]^+^	334 (352)	20	20.2 (0.9)	4.2	8.4 (2.7)
			40	41.1 (1.5)	3.6	
			320	330.4 (27.1)	8.2	
FB_2_	706 [M + H]^+^	336 (318)	20	18.3 (4.0)	21.7	6.8 (2.2)
			40	38.8 (5.2)	13.5	
			320	338.4 (23.7)	7.0	
AFB_1_	313 [M + H]^+^	285 (128)	1	0.98 (0.1)	10.4	0.5 (0.1)
			2	2.04 (0.16)	7.8	
			16	16.3 (0.39)	2.4	
AFB_2_	315 [M + H]^+^	287 (259)	1	1.02 (0.08)	7.6	0.4 (0.1)
			2	2.07 (0.11)	5.0	
			16	15.8 (0.79)	5.1	
AFG_1_	329 [M + H]^+^	243 (200)	1	1.04 (0.12)	11.2	0.5 (0.2)
			2	2.05 (0.13)	6.2	
			16	15.9 (1.01)	6.4	
AFG_2_	331 [M + H]^+^	313 (245)	1	1.1 (0.10)	9.5	1.0 (0.4)
			2	2.12 (0.11)	5.3	
			16	15.7 (0.4)	2.5	

^1^ Calculated concentrations (±SD) for each calibration level represent the accuracy of measurements; ^2^ and the relative standard deviation (RSD) represents the repeatability.

**Table 5:** Mycotoxin contamination in stored wheat grain samples (ppb).

Sample #	AFB_1_	AFB_2_	AFG_1_	AFG_2_	FB_1_	FB_2_	DON	OTA	T-2	ZEN
1–3	-	-	-	-	-	-	-	-	-	-
4	2.1	-	-	-	179.4	13.3	16.7	-	-	-
5	-	-	-	-	6.2	2.2	-	-	-	-
6	-	-	-	-	428.4	24.5	1.2	-	-	0.7
7	-	-	-	-	4.3	-	-	-	-	-
8	-	-	-	-	-	-	-	-	-	-
9	-	-	-	0.2	-	-	2.8	-	-	-
10	-	-	-	-	-	-	-	-	-	-
11	-	-	-	-	-	-	4.6	-	-	-
12–17	-	-	-	-	-	-	-	-	-	-
18	-	-	-	1.1	2340.7	87.9	-	-	-	-
19	-	-	-	0.3	53.9	4	-	-	-	-
20	6.2	-	-	0.4	62.6	4.8	-	-	-	8.9
21	5.2	-	-	0.6	135.1	11.8	-	1.9	-	3.8
22	-	-	-	-	-	-	263.5	-	-	7.6
23	-	-	-	-	-	-		-	-	-
24	-	-	-	-	-	-	1746.5	-	-	64.8
25	-	-	-	-	-	-	-	-	-	-
26	32.3	1.8	-	-	106.7	7.5	-	-	-	-
27	-	-	-	-	-	-	-	-	-	-
28	-	-	-	-	-	-	8.1		-	-
29	-	-	-	-	-	-	-	22.8	-	-
30	-	-	-	-	-	-	-	41.5	-	-
31	-	-	-	-	-	-	5.7	-	-	-
32–34	-	-	-	-	-	-	-	-	-	-

"-" not detected.

## Supplementary Materials

The following are available online at www.mdpi.com/2072-6651/9/10/302/s1, Figure S1: Species specific multiplex PCR assays using DNA isolated from stored wheat grain, Table S1: Primer sets used in this study (including references [53,54,55,56,57,58,59,60,61,62,63,64,65,66,67,68,69,70,71,72]), Table S2: Fungal isolates used in this study.
