# Supplementary material for: Rapid Detection and Identification of Mycotoxigenic Fungi and Mycotoxins in Stored Wheat Grain
Source: Toxins (Basel). 2017 Sep 25;9(10):302. doi: 10.3390/toxins9100302 (PMC5666349; doi:10.3390/toxins9100302)
Supplement: Supplementary file 1 [file toxins-09-00302-s001.pdf]

Supplementary Materials: Rapid Detection and Identification of Mycotoxigenic Fungi and Mycotoxins in Stored Wheat Grain

Sudharsan Sadhasivam, Malka Britzi, Varda Zakin, Moshe Kostyukovsky, Anatoly Trostanetsky, Elazar Quinn and Edward Sionov

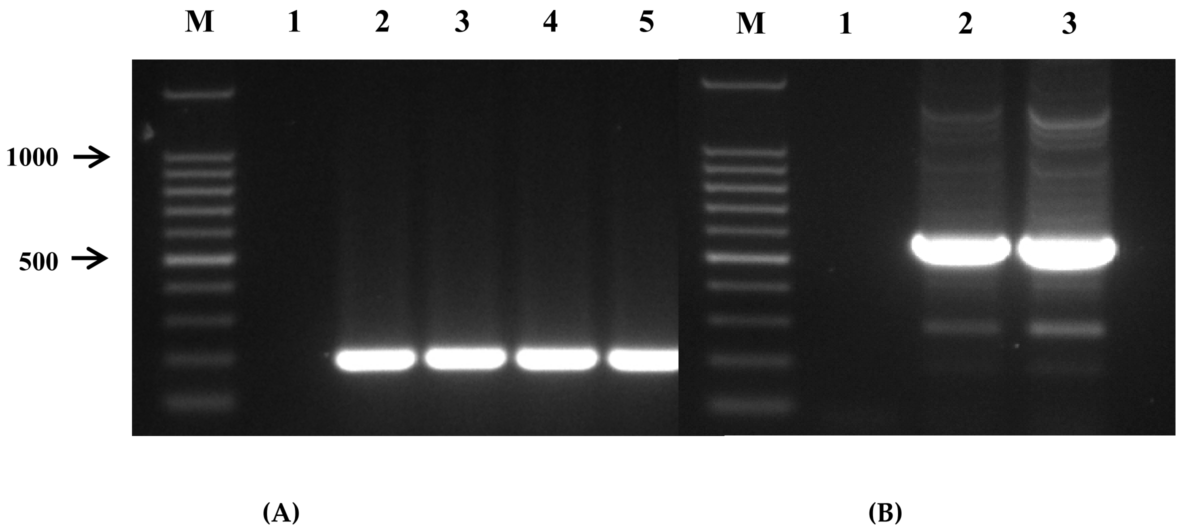

Figure S1. Species specific multiplex PCR assays using DNA isolated from stored wheat grain. (**A**) Primer set I (Aspergillus species-specific primers). Lanes: M – 100bp DNA ladder; 1 – Negative control; 2-5 – wheat grain samples naturally contaminated by A. flavus; (**B**) Primer set II (Fusarium species-specific primers). Lanes: 1 – Negative control; 2, 3 – wheat grain samples naturally contaminated by F. culmorum.

Table S1. Primer sets used in this study

Set no.	Species	Primer name	Sequence (5'-3')	Size (b/p)	Annealing temperature	References
I	*A. fumigatus*	PEX1	TATGTCTTCCCCTGCTCC	250	60 °C	Logotheti *et al.*, 2009 [53]
		PEX2	CTATGCCTGAGGGGCGAA			
	A. flavus	PepO1	CGACGTCTACAAGCCTTCTGGAAA	200		Logotheti *et al.*, 2009 [53]
		PepO2	CAGCAGACCGTCATTGTTCTTGTC			
	A. parasiticus	PAR1	GTCATGGCCGCCGGGGGCGTC	430		Sardinas *et al.*, 2010 [31]
		PAR2	CCTGGAAAAAATGGTTGTTTTGCG			
	A. tubingensis	TUB1	TCGACAGCTATTTCCCCCTT	505		Susca *et al.*, 2007 [54]
		TUB2	TAGCATGTCATATCACGGGCAT			
	A. carbonarius	CARBO1	AAGCGAATCGATAGTCCACAAGAATAC	371		Perrone *et al.*, 2004 [55]
		CARBO2	TCTGGCAGAAGTTAATATCCGGTT			
II	*F. graminearum*	Fg16F	CTCCGGATATGTTGCGTCAA	400-500	55 °C	Nicholsons *et al.*, 1998 [56]
		Fg16R	GGTAGGTATCCGACATGGCAA			
	F. culmorum	Fc01F	ATGGTGAACTCGTCGTGGC	570		Nicholsons *et al.*, 1998 [56]
		Fc01R	CCCTTCTTACGCCAATCTCG			
	F. poae	Fp82F	CAAGCAAACAGGCTCTTCACC	220		Parry and Nicholsons, 1996 [57]
		Fp82R	TGTTCCACCTCAGTGACAGGTT			
	F. sporotrichioides	AF330109CF	AAAAGCCCAAATTGCTGATG	332		Demeke *et al.*, 2005 [58]
		AF330109CR	TGGCATGTTCATTGTCACCT			
III	*F. verticillioides*	VER1	CTTCCTGCGATGTTTCTCC	578	55 °C	Mule *et al.*, 2004 [59]
		VER2	AATTGGCCATTGGTATTATATAC			
	F. avenaceum	FaF	CAAGCATTGTCGCCACTCTC	920		Doohan *et al.*, 1998 [60]
		FaR	GTTTGGCTCTACCGGGACTG			
	F. solani	FS1	GCAGGTATGGCTTTTTGGAA	175		Casasnovas *et al.*, 2013 [61]
		FS2	AGTAAACTCCGACAGGTGCAA			
	A. niger	An F	GATTTCGACAGCATTTTCCAGAA	357		Palumbo *et al.*, 2015 [62]
		An R	GATAAAACCATTGTTGTCGCGGTCG			
IV	*F. proliferatum*	PRO1	CTTTCCGCCAAGTTTCTTC	585	55°C	Mule *et al.*, 2004 [59]
		PRO2	TGTCAGTAACTCGACGTTG			
	F. oxysporum	FOF1	ACATACCACTTGTTGCCTCG	340		Mishra *et al.*, 2003 [63]
		FOR1	CGCCAATCAATTTGAGGAACG			
	P. expansum	PE1	AATGTGTACTGACTGGTCGCAG	480		Dombrink-Kurtzman & Mcgovern, 2007 [64]
		PE2	CAACCAACATATTCGTGCCTGAC			
	P. digitatum	Pri 207	TAGCTCCAAAACAAATCGTCTGGC	250		Hamamoto *et al.*, 2000 [65]
		Pri 38c	CACTTGATCTGCCCTGTTAACA			
V	*P. paneum*	PP1	GAATACACACTGACTGGC	482	60°C	Dombrink-Kurtzman & Mcgovern, 2007 [64]
		PP2	TCAACCAACACATTCGTACCAGAC			

	P. verrucosum	otanpsF<br>otanpsR	AGTCTTCGCTGGGTGCTTCC<br>CAGCACTTTTCCCTCCATCTATCC	750		Bogs *et al.*, 2006 [66]
	P. roqueforti	ITS183<br>ITS401	CTGTCTGAAGAATGCAGTCTGAGAAC<br>CCATACGCTCGAGGACCGGAC	300		Pedersen *et al.*, 1997 [67]
	A. terreus	ATRF81<br>ATRR120	TACCTTCAAGCCTGACTACG<br>ACCTGCTCGGCCAGTTTGCTG	386		Kanbe *et al.*, 2002 [68]
**Set no.**	**Mycotoxin**	**Primer**	**Sequence (5'-3')**	**Size**	**Annealing temperature**	**References**
VI	Aflatoxins	aflR1F<br>aflR1R	AACCGCATCCACAATCTCAT<br>AGTGCAGTTCGCTCAGAACA	798	58°C	Manonmani *et al.*, 2005 [69]
		NorF<br>NorR	ACCGCTACGCCGGCGCTCTCGGCAC<br>GTTGGCCGCCAGCTTCGACACTCCG	397		Priyanka *et al.*, 2014 [70]
		avf723F<br>avf1675R	ATGGTCACATACGCCCTCCTCGGG<br>GCCTCGCATTCTCTCGGCGACCGAA	950		Yu *et al.*, 2000 [71]
		ver1<br>ver2	GCCGCAGGCCGCGGAGAAAGGTGGT<br>CCGCAGTCAATGGCCATGCAGCG	452		Skory *et al.*, 1992 [72]
VII	Fumonisin/ Trichothecene/ Zearalenone	Fum1F<br>Fum1R	ATTATGGGCATCTTACCTGGAT<br>ACGCAAGCTCCTGTGACAGA	798	55°C	Ramana *et al.*, 2011 [11]
		Fum13F<br>Fum13R	AGTCGGGGTCAAGAGCTTGT<br>TGCTGAGCCGACATCATAATC	988		Ramana *et al.*, 2011 [11]
		tri5F<br>tri5R	GAGAACTTTCCCACCGAATAT<br>GATAAGGTTCAATGAGCAGAG	450		Ramana *et al.*, 2011 [11]
		tri6F<br>tri6R	GATCTAAACGACTATGAATCACC<br>GCCTATAGTGATCTCGCATGT	546		Ramana *et al.*, 2011 [11]
		ZEA13F<br>ZEA13R	CATTCTTGGTCTTGTGAGGA<br>CCTTATGCTCATCGACATG	351		Priyanka *et al.*, 2015 [8]
VIII	Ochratoxin A	Aolc35F<br>Aolc12R	GCCAGACCATCGACACTGCATGCTC<br>CGACTGGCGTTCCAGTACCATGAGC	536	58°C	Priyanka *et al.*, 2015 [8]
		otanpsF<br>otanpsR	AGTCTTCGCTGGGTGCTTCC<br>CAGCACTTTTCCCTCCATCTATCC	750		Bogs *et al.*, 2006 [66]

Table S2. Fungal isolates used in this study

**Standard strains**
A. carbonarius NRRL 368
A. flavus NRRL3518
A. fumigatus NRRL 62427
A. niger NRRL 328
A. parasiticus NRRL6111
A. terreus NRRL 269
A. tubingensis NRRL 66281
A. ochraceus NRRL 35018
F. avenaceum NRRL A-28073
F. culmorum NRRL 13320
F. graminearum NRRL 3376
F. poae NRRL 36300
F. proliferatum NRRL 31866
F. roseum NRRL 6469
F. solani NRRL 13416
F. sporotrichioides NRRL3299
F. verticillioides NRRL 25457
P. digitatum NRRL 1202
P. expansum NRRL 976
P. roqueforti NRRL 849
P. verrucosum NRRL 965
P. viridicatum NRRL 5571
**Fungal strains isolated from wheat grains**
A. flavus SS1
A. flavus SS2
A. fumigatus SS3
F. verticillioides SS4
F. verticillioides SS5
F. culmorum SS6
P. viridicatum SS7
M. ruber SS8
